# Supplementary material for: Iterative improvement in the automatic modular design of robot swarms
Source: PeerJ Comput Sci. 2020 Dec 7;6:e322. doi: 10.7717/peerj-cs.322 (PMC7924708; doi:10.7717/peerj-cs.322)
Supplement: Supplemental Information 3 [file peerj-cs-06-322-s003.zip › argos3/doc/api/standalone/a00599_source.html]

ARGoS: plugins/simulator/media/rab\_medium.h Source File


- Main Page
- Related Pages
- Namespaces
- Classes
- Files

- File List
- File Members

# plugins/simulator/media/rab\_medium.h

Go to the documentation of this file.

```
00001 #ifndef RAB_MEDIUM_H
00002 #define RAB_MEDIUM_H
00003 
00004 namespace argos {
00005    class CRABMedium;
00006    class CRABEquippedEntity;
00007 }
00008 
00009 #include <argos3/core/simulator/medium/medium.h>
00010 #include <argos3/core/simulator/space/positional_indices/positional_index.h>
00011 #include <argos3/plugins/robots/generic/control_interface/ci_range_and_bearing_sensor.h>
00012 #include <argos3/plugins/simulator/entities/rab_equipped_entity.h>
00013 
00014 namespace argos {
00015 
00016    class CRABMedium : public CMedium {
00017 
00018    public:
00019 
00023       CRABMedium();
00024 
00028       virtual ~CRABMedium();
00029 
00030       virtual void Init(TConfigurationNode& t_tree);
00031       virtual void PostSpaceInit();
00032       virtual void Reset();
00033       virtual void Destroy();
00034       virtual void Update();
00035 
00040       void AddEntity(CRABEquippedEntity& c_entity);
00041       
00046       void RemoveEntity(CRABEquippedEntity& c_entity);
00047 
00054       const CSet<CRABEquippedEntity*>& GetRABsCommunicatingWith(CRABEquippedEntity& c_entity) const;
00055 
00056    private:
00057 
00059       typedef std::map<CRABEquippedEntity*, CSet<CRABEquippedEntity*> > TRoutingTable;
00060 
00062       TRoutingTable m_tRoutingTable;
00063 
00065       CPositionalIndex<CRABEquippedEntity>* m_pcRABEquippedEntityIndex;
00066 
00068       CRABEquippedEntityGridEntityUpdater* m_pcRABEquippedEntityGridUpdateOperation;
00069 
00070    };
00071 
00072 }
00073 
00074 #endif
```

---

Generated on 10 Jul 2018 for ARGoS by 
 1.6.1
